# On the fluid dynamics of a laboratory scale single-use stirred bioreactor

**DOI:** 10.1016/j.ces.2014.02.032

**Published:** 2014-05-24

**Authors:** A.O.O. Odeleye, D.T.J. Marsh, M.D. Osborne, G.J. Lye, M. Micheletti

**Affiliations:** a Department of Biochemical Engineering, University College London, Torrington Place, London WC1E 7JE, United Kingdom; b Eli Lilly S.A. Irish Branch, Dunderrow, Kinsale, Co. Cork, Ireland

**Keywords:** Bioreactors, Hydrodynamics, Mammalian Cell Culture, PIV, Scale-up, Single-use

## Abstract

The commercial success of mammalian cell-derived recombinant proteins has fostered an increase in demand for novel single-use bioreactor (SUB) systems that facilitate greater productivity, increased flexibility and reduced costs (Zhang et al., 2010). These systems exhibit fluid flow regimes unlike those encountered in traditional glass/stainless steel bioreactors because of the way in which they are designed. With such disparate hydrodynamic environments between SUBs currently on the market, traditional scale-up approaches applied to stirred tanks should be revised. One such SUB is the Mobius^®^ 3 L CellReady, which consists of an upward-pumping marine scoping impeller. This work represents the first experimental study of the flow within the CellReady using a Particle Image Velocimetry (PIV) approach, combined with a biological study into the impact of these fluid dynamic characteristics on cell culture performance. The PIV study was conducted within the actual vessel, rather than using a purpose-built mimic. PIV measurements conveyed a degree of fluid compartmentalisation resulting from the up-pumping impeller. Both impeller tip speed and fluid working volume had an impact upon the fluid velocities and spatial distribution of turbulence within the vessel. Cell cultures were conducted using the GS-CHO cell-line (Lonza) producing an IgG_4_ antibody. Disparity in cellular growth and viability throughout the range of operating conditions used (80–350 rpm and 1–2.4 L working volume) was not substantial, although a significant reduction in recombinant protein productivity was found at 350 rpm and 1 L working volume (corresponding to the highest Reynolds number tested in this work). The study shows promise in the use of PIV to improve understanding of the hydrodynamic environment within individual SUBs and allows identification of the critical hydrodynamic parameters under the different flow regimes for compatibility and scalability across the range of bioreactor platforms.

## Highlights

• PIV is used to quantify fluid dynamics in a small scale rigid single-use bioreactor.
• The influence of the hydrodynamic environment upon CHO cells was studied.
• Modest impact upon cell viability and viable cell density is observed.
• Distinct shifts in metabolic behaviour along with IgG_4_ productivity is noted.

## 1 Introduction

In the last 15 years the biopharmaceutical industry has experienced a significant increase in the uptake and utilisation of single-use technologies, primarily driven by the need for a reduced time to market, an enhanced degree of flexibility and attenuated risk of cross-contamination (Business Insights, 2007). Since the release of the first rocked bag by Wave Biotech US in 1996 (GE Healthcare Life Sciences), several single-use bioreactor (SUB) products have become available for upstream processing, and in particular, for cell culture applications. Such systems typically consist of a multilayer polymer bag mounted on a metal skid, where polypropylene is often used as the contact layer (Barbaroux and Sette, 2006), or of a rigid plastic case moulded to the desired shape and geometry. SUBs can be distinguished by their mixing mechanism and/or bioreactor geometry. There are bag-based, stirred tank and hollow-fibre bioreactors (Eibl et al., 2009), mechanically (i.e. tipping, stirring or vibrating) or pneumatically driven devices such as airlift or slug bubble devices (Terrier et al., 2006). The 3 L CellReady was one of the first rigid stirred tank SUBs to be released on the market and has been widely employed for process development as its geometrical configuration facilitates process translation to larger scales of operation. It is part of EMD Millipore׳s family of Mobius^®^ CellReady SUBs, including 50 L and 200 L vessels suitable for pilot-scale and clinical-scale applications. Recently, a few single-use equipment manufacturers have invested in larger scale systems (examples include the 500 L Pneumatic Bioreactor System and 500 L GE WAVE Bioreactor^™^). However, greater process predictability and robust scale translation methods must be ensured if laboratory scale single-use bioreactor systems are to be used as scale-down models at the development stage.

Mammalian cells, due to their capacity for assembly, correct protein folding and post-translational modifications, have become the dominant host cell type for the production of therapeutic proteins for clinical use in humans. Monoclonal antibodies in particular represent an important class of therapeutics whose benefits to patients have been recognised in the fields of oncology and immunology (Pavlou and Reichert, 2004; Reichert et al., 2005). Commercial production of these antibodies relies on the development of a robust large scale cultivation process step. While SUBs represent the cost-effective choice for cell cultivation, to date the ability to optimise and translate the process to larger scales has been rather limited. Rigorous fluid dynamics studies and the definition of appropriate scaling parameters in novel SUB systems are crucial to improve understanding of the effect of the hydrodynamic environment on cellular performance, and to ensure the same process and product characteristics are achieved at different scales in line with regulatory demands and Quality by Design approaches.

The importance of a well-mixed environment and the effect of operating conditions on cell growth and productivity have been widely documented (Abu-Reesh and Kargi, 1991). High agitation rates in a stirred tank bioreactor were found to significantly impact upon cell viability, glucose consumption rates and MAb production of hybridoma cells grown in 15% serum medium (Abu-Reesh and Kargi, 1991). A few studies have indicated that cells which are acclimated to high agitation rates are more resistant to the hydrodynamic stresses associated with the impeller rotation than those that are suddenly exposed to an increase in turbulence levels (Petersen et al., 1988; Schmid et al., 1992). In addition, cell physiology plays an important role as cells were found to respond differently to an increase in impeller agitation according to their growth stage (Petersen et al., 1990, 1988). The combination of air flow rate and increasing impeller rates of up to 300 rpm have been found to cause a decrease in stationary phase cell viability of TB/C3 hybridoma from over 95% to approximately 75% (Oh et al., 1989). In addition, Sorg et al. (2011) have demonstrated that the environmental heterogeneity of shear stress is as important as the mean stress that cells experience along their path. In their work, a Lobed Taylor-Couette bioreactor was used to simulate the hydrodynamic stress conditions occurring in the impeller zone of a stirred tank reactor, with estimated mean shear stress values of up to 0.4 Pa. An increase in lactate production and a decrease in antibody titre was observed, whilst consumption of the primary nutrients remained largely unchanged.

Although one of the major operational issues with regards to mammalian cell culture is the cellular response to shear forces (Abu-Reesh and Kargi, 1991; Cherry, 1993; Kretzmer and Schiigerl, 1991), it is generally noted that mammalian cells can physically tolerate the typical hydrodynamic stresses induced by the impeller within stirred tank bioreactors (Oh et al., 1989). The primary cause of damage has been attributed to interfacial shear and therefore to air bubble breakage and coalescence during culture (Ma et al., 2002). The aim for many of the novel mixing regimes designed into SUBs is to provide an environment that further enhances cellular productivity, whilst maintaining mixing performance for optimal cell growth.

The measurement of fluid dynamic characteristics in three-dimensional (3-D) turbulent flows is highly challenging, due to the space–time variation of turbulence levels and energy dissipation rates. The presence of a gas phase enhances the system complexity (Aubin et al., 2004). There have been a number of studies using laser-based techniques to obtain velocity field information within stirred tank reactors (Baldi et al., 2002; Deen and Hjertager, 2002; Ducci and Yianneskis, 2005; Gabriele et al., 2009; Hill et al., 2008; Pan et al., 2008 to name a few). Using both time-resolved and phase-resolved measurements, characteristics such as turbulent kinetic energy (TKE), shear stress and estimates of the rate of viscous dissipation of turbulent kinetic energy (*ε*) can be determined. However, two-dimensional (2-D) imaging techniques have inevitable resolution limitations depending on the camera field of view and lens properties, as well as dimensional restrictions, making assumptions necessary (Khan, 2005). Spatial fluctuating velocity gradients can be used to obtain an estimate of the rate of viscous dissipation of TKE (Hinze, 1975), however, consideration must be given to inaccuracies that arise from calculating such rates at length scales below the actual measurement resolution (Gabriele et al., 2009). It has been demonstrated that techniques such as the Smagorinsky Closure Method can be useful in estimating *ε* at such scales with a reasonable degree of accuracy (Meyers and Sagaut, 2006).

Despite the need for detailed information on velocity and mixing characteristics in single-use bioreactors, few studies have focused on the engineering characterisation of these novel devices (Löffelholz et al., 2013; Nienow et al., 2013a). Kaiser et al. (2011) used Computational Fluid Dynamic (CFD) approaches to determine engineering characteristics such as mixing time, power input and oxygen transfer within the 3 L Mobius^®^ CellReady bioreactor. The marine impeller fitted within this bioreactor was found to produce an up-pumping circulation loop directed 25° above the horizontal plane, with the fluid velocity dominated by its radial component. In addition, gas distribution was found to be significantly heterogeneous, with flooding of the impeller observed up to an impeller rate of *N*=200 rpm (*Re*=21,747). A stagnant zone characterised by negligible average velocity values was also observed at the drain inlet, leading to cell sedimentation and accumulation in the region (Kaiser et al., 2011). The Euler–Euler approach was employed to simulate multiphase flow in the work of Kaiser et al. (2011). However, as a result of assuming uniform bubble size, a variation of approximately 40% was noted between CFD predictions and measured values of the gas liquid mass transfer coefficient in the bioreactor. While several fluid dynamic investigations have utilised Particle Image Velocimetry (PIV) approaches to obtain flow pattern information, velocity field and local energy dissipation rates in a stirred tank reactor geometry, in most cases results were obtained using a reactor mimic with a standard geometry configuration as opposed to an actual commercially available bioreactor. Furthermore, the impact of these experimentally obtained whole-field flow characteristics upon cellular behaviour has rarely been investigated at working conditions. This work aims to carry out a rigorous fluid dynamic study of the flow within a novel single-use bioreactor (3 L Mobius^®^ CellReady) in order to improve understanding of its flow pattern, mixing efficiency and velocity characteristics. PIV experimentation was conducted using a model fluid made of water and 10 μm particles under un-gassed conditions, to prevent laser refraction caused by the gas phase within the liquid. Although this is not ideal, PIV studies have shown the peak turbulence levels measured in the impeller discharge zone, to remain stable between aerated and non-aerated conditions (Aubin et al., 2004; Zhu et al., 2009). The acquired hydrodynamic knowledge was subsequently used to investigate the impact of operating conditions on the cell culture performance of a Chinese Hamster Ovary (CHO) mammalian cell-line.

## 2 Materials and methods

### 2.1 Bioreactors configuration

**Fig. 1:**
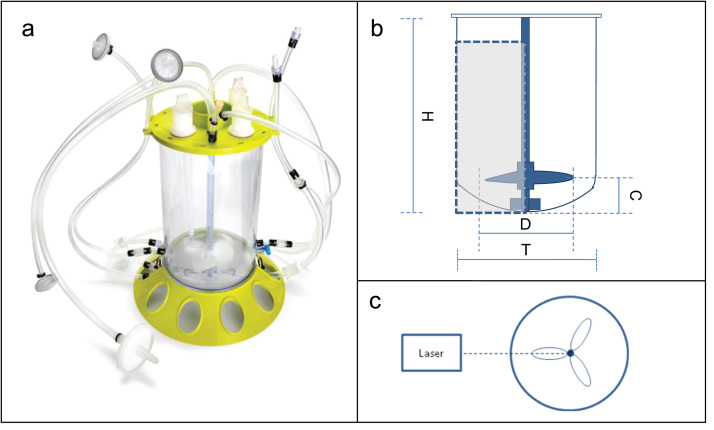
(a) Mobius^®^ 3 L CellReady Bioreactor. Image from www.millipore.com, accessed 17.02.2012; (b) schematic diagram of bioreactor where the shaded area indicates the measurement plane; and (c) top-down view of bioreactor and laser.

The bioreactor employed in the PIV measurements and cell culture experiments is the Mobius^®^ 3 L CellReady. It is an unbaffled stirred tank made of polycarbonate, marketed by Merck Millipore for cell culture process development experimentation. Equipped with a three-blade marine scoping impeller, the vessel is available pre-assembled and sterilised via gamma irradiation. Fig. 1 displays an image of the 3 L Mobius^®^ CellReady (Fig. 1a), a schematic representation of the bioreactor with the measurement area shaded (Fig. 1b) and a horizontal top-down view of the laser and CellReady system (Fig. 1c). Cell culture experiments were also conducted using a Sartorius B.Braun BIOSTAT^®^ B-DCU 5 L bioreactor. The bioreactor has a 3.5 L working volume (5 L total volume) and it is stirred by a three-blade segment pitched (45°) impeller. The dimensions of both bioreactors and their impellers are listed in Table 1.

### 2.2 PIV system and data processing

A cylindrical coordinate system is used, with the radial, axial and azimuthal coordinates indicated in this work by *r*, *z* and *θ* respectively. The system׳s origin is located in the centre of the bioreactor bottom and *θ*=0° corresponds to the vertical plane intersecting the centre of the blade and aligned to the shaft. The chosen laser orientation will allow for the acquisition of velocities in the axial and radial directions. The importance of the tangential component of velocity is recognised, however, obtaining velocities in the tangential direction would be difficult without removing the curved bioreactor base. Therefore, this component of velocity was not measured in the present study. The CellReady bioreactor was enclosed within a square glass tank filled with RO water, in order to minimise errors due to refraction/diffraction of the laser on the cylindrical surface. The bioreactor was also filled with RO water and seeded with 10 µm silvered hollow glass spheres. RO water was deemed appropriate as a model fluid because the glucose concentration of CD-CHO media and the cell density attained during cell culture would not significantly increase the culture viscosity above that of water (Chimica et al., 1999; Clincke et al., 2013).

A Dantec Dynamics PIV system was employed to measure velocity characteristics. Measurements were carried out for a range of impeller speeds *N*=80–350 rpm, corresponding to Reynolds numbers (*Re*) of 8699–38057, at constant bioreactor fill volume equal to 2.4 L. Experiments were also performed at fluid working volumes of 1, 1.8 and 2.4 L at a constant impeller speed of *N*=200 rpm (*Re*=21,747). Two 50 mJ DualPower 50-100 (Nd:YAG) lasers were employed to provide a light sheet at a wavelength of 532 nm. A timer box, an Allen-Bradley (Ultraware 3000) motor, encoder and the trigger system along with 4×10 Bayonet Neill-Concelman connectors (BNC) were used to synchronise the laser pulses and trigger a SpeedSense 1020 camera. Images were taken in the vertical plane in correspondence to the impeller shaft as shown in Fig. 1b. The DynamicStudio software was used to process the raw images captured by the camera. Each velocity vector field was determined from an adaptive correlation with 50% overlap and a final interrogation area of 16×16 pixels. The lowest spatial resolution attained within a vector map was 0.53×0.53 mm.

Both time and phase-resolved measurements were acquired. In order to carry out phase-resolved measurements the camera and laser were synchronised to the rotation rate of the impeller, and set to collect images once per revolution at the required angular position. Angular positions from *θ*=0° to 105° were investigated every 15° at different rotational speeds and for varying fill volumes, where an angle of *θ*=0° corresponds to the vertical laser light sheet intersecting the mid-point of the leading impeller. For each condition 250–500 image pairs were taken to produce 250–500 instantaneous velocity vector maps. Root-mean-square velocity values for both 250 and 500 vector maps were compared in order to test statistical differences, resulting in an average error of less than 1%. These were then post-processed using a MATLAB program to determine different fluid dynamic characteristics using the mean, instantaneous and turbulent velocity components.

### 2.3 Cell culture methods

A Glutamine Synthetase Chinese Hamster Ovary (GS-CHO) cell-line expressing IgG_4_ B72.3 (Lonza Biologics, Slough, UK) was used for the cell culture experiments. Cells were grown in Chemically Defined (CD) CHO medium (Invitrogen, Paisley UK). The inoculum was prepared in disposable vented cap shake flasks and incubated using a Galaxy S incubator (Wolf Laboratories, York, UK) at 37 °C, 5% (v/v) CO_2_ producing 10% of the total cell culture volume. In the experiments conducted in the CellReady bioreactor, pH was controlled at 7 and dissolved oxygen tension (DOT) was set to a minimum of 30% and controlled using carbon dioxide and 100 mM sodium bicarbonate. The airflow rate was set constant at 0.1 vvm. A solution of 10× concentrated CD-CHO medium with 150 g/L of glucose was used to feed the cells once the glucose concentration of the cell culture dropped below 2 g/L. Cell culture experiments were performed at different impeller speeds and liquid volumes, as shown in Table 2.

Cell culture experiments in the Sartorius 5 L BIOSTAT^®^ bioreactor were conducted at *N*=260 rpm (corresponding to *Re=*23,858). Air flow rate was maintained constant at 0.02 vvm and the DOT was controlled at a minimum of 30%. The *k*_*L*_*a* value, as measured via the dynamic gassing-out method, was found to be 5 hr^−1^ at the operating conditions used in this work. The BIOSTAT^®^ cell culture was used as a benchmark for culture performance.

### 2.4 Analytical techniques

Samples withdrawn from the CellReady and BIOSTAT^®^ bioreactors were analysed for viable cell concentration and cell viability (trypan blue exclusion method) using a ViCell (Beckman Coulter, High Wycombe, UK). A CASY analyser (Innovatis, Bielefeld, Germany) was used to determine the cell size distribution. For each condition measurements were repeated 5 times and the mean and standard deviation values calculated. The cell size frequency was normalised by the number of counts associated with the peak cell diameter frequency. The measured cell diameter ranges were set at 13.5–40 µm and 7.5–13.5 µm for viable and non-viable cells, respectively. Cell culture samples were centrifuged at 13,200 rpm for 10 min in a Microcentrifuge 5415 R system (Eppendorf North America, USA) in order to separate the cells from the supernatant. A Nova Bioprofile 400 Analyser (Nova Biomedical, Waltham, MA, USA) was used to measure the concentration of metabolites present in the sample supernatant, including glutamine, glutamate, glucose and lactate, as well as ammonium, sodium and potassium ion concentrations and osmolality.

The monoclonal antibody IgG_4_ concentration was determined by protein G-HPLC analysis using an Agilent 1200 HPLC system (Agilent Technologies, South Queensferry, UK). 200 µL samples were prepared and loaded onto a 96-well plate (diluted 1 to 2 in 20 mM Sodium Phosphate). 100 µl of the sample was injected in to a 1mL HiTrap protein G column (GE Healthcare, Pittsburgh, PA) and washed with 20 mM sodium phosphate buffer (at pH 7), with the analysis being performed at a flow rate of 1mL/min. Samples were eluted using a glycine buffer (20 mM, adjusted to pH 2.8) and the elution peak was measured by UV detection at 280 nm. The peak corresponding to the IgG_4_ (at 5.7 min) was integrated and the antibody concentration determined using a standard calibration curve. Cell specific daily productivity was determined using the integral viable cell concentration (IVC), as shown in the equation below (Smales and James, 2005).

(1) $${\mathrm{IVC}}_{i+1}=0.5(C_{i+1}+C_{i})(t_{i+1}-t_{i})/24+{\mathrm{IVC}}_{i}$$

A plot of IgG_4_ concentration (pg/mL) vs. IVC (cells day/mL) was produced, and a linear curve fitted to determine the cell specific productivity (pg/cell/day).

## 3 Results and discussion

### 3.1 Whole field flow characteristics

**Fig. 2:**
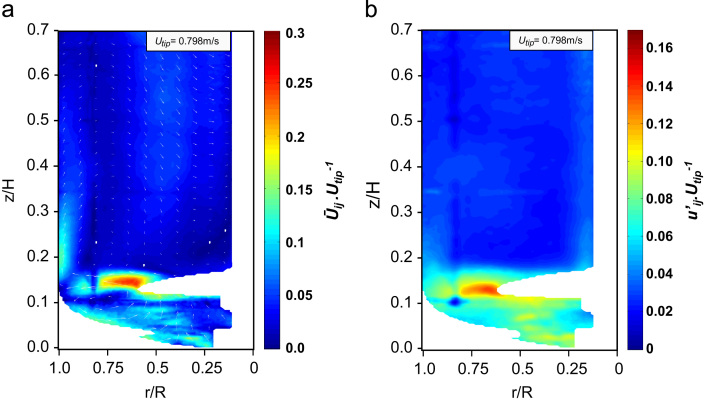
(a) Ensemble-averaged velocity magnitude and contour plot (*V*_*L*_=2.4 L and *N*=200 rpm); (b) r.m.s of the fluctuating velocity component (in axial and radial directions) contour plot (*V*_*L*_=2.4 L and *N*=200 rpm).

A description of the single-phase flow occurring inside the stirred Mobius CellReady bioreactor is provided in this section. In the first part, the analysis is carried out for a standard configuration corresponding to a fill volume of 2.4 L and an impeller rotational speed of 200 rpm (*Re*=21,747). In the subsequent section this analysis is extended to phase-resolved data (at *N*=120 rpm, *Re*=13,048) as well as to a range of impeller rotational speeds and liquid fill volumes. A Particle Image Velocimetry (PIV) system, described in Section 2, was used to obtain time-resolved, phase-resolved and ensemble-averaged velocity data. The two-dimensional (2-D) ensemble-average velocity field (with vectors superimposed) and contour plot of the r.m.s. of turbulent velocity are shown in Fig. 2. Fig. 2a shows the impeller inducing an upward flow at a trajectory of approximately 26° with respect to the horizontal plane, the fluid then impinges on the wall at a vertical position of *z*/*H*=0.15, at which point the fluid divides into two counter-rotating vortices in the regions below and above the impeller. The vortex located below the impeller plane is characterised by ensemble-averaged velocities of up to 0.25*U*_*tip*_. This value is significantly lower than the 0.55*U*_*tip*_ measured by Gabriele et al. (2009) using an up-pumping 45° pitched blade turbine, however the value obtained in this work is in good agreement with the 0.26*U*_*tip*_ measured by Baldi et al. (2002) for a down-pumping 3-bladed hydrofoil axial impeller (*D*/*T*=0.33 and a trailing edge angle of approximately 10°) and with the 0.25*U*_*tip*_ measured by Plion et al. (1985) using a propeller. In the CellReady bioreactor the top circulation loop is weaker and achieves approximately a height of *z*/*H*=0.5 before turning downwards towards the impeller region. The regions in correspondence with the vortex centre and on top of the vessel above *z*/*H*=0.5 are characterised by a limited degree of mixing and turbulence levels below 0.05*U*_*tip*_ (Fig. 2b). The maximum velocity of 0.25*U*_*tip*_ is achieved in the impeller discharge stream (at approximately *r*/*R*=0.65 and *z*/*H*=0.15), whilst the r.m.s. of the fluctuating velocity components in the axial and radial directions $\left(u_{j}^{\prime },\;u_{i}^{\prime }\right)$ are 0.15*U*_*tip*_ and 0.11*U*_*tip*_ respectively. These values are in good agreement with the corresponding r.m.s. values of 0.12*U*_*tip*_ and 0.15*U*_*tip*_, respectively, obtained by Aubin et al. (2004) in the fluid discharge stream of an upward pumping 45° pitched blade turbine (*D*=0.5*T*). The contour plot in Fig. 2a was purposely made to allow comparison with the CFD simulations of Kaiser et al. (2011), which is the only work available in literature that provides a qualitative study of the flow in the 3 L Mobius^®^ CellReady impeller stream. The current results are in good agreement with their simulations, characterised by maximum velocities of 0.25*U*_*tip*_ at an impeller speed of *N*=200 rpm for a fluid of similar viscosity. The presence of a circulation loop occupying the bottom third of the tank has also been reported by Kaiser et al. (2011) simulations. Considerable difference of up to 80% between the impeller zone and bulk fluid turbulence levels can also be observed from Fig. 2b.

**Fig. 3:**
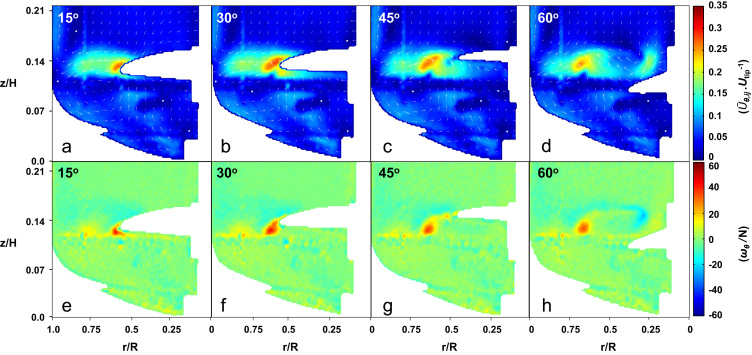
(a–d) Contour plots of the phase-resolved velocity magnitude at *N*=120 rpm with velocity vectors superimposed at angles: (a) *θ*=15°, (b) *θ*=30°, (c) *θ*=45°, and (d) *θ*=60°. (e–h) Contour plots of the phase resolved tangential vorticity (blue and red indicating clockwise and anti-clockwise rotation respectively) at *N*=120 rpm and angles: (e) *θ*=15°, (f) *θ*=30°, (g) *θ*=45°, and (h) and *θ*=60°.

The 2-D phase-resolved velocity fields (${\overset{¯}{U}}_{\theta ,ij}/U_{tip}$) and contour plots of the vorticity around the tangential axis (*ω*_*θ*_/*N*) are shown in Fig. 3 for four phase angles at *N*=120 rpm (*Re*=13,048). Periodic fluctuations in velocity fields and vorticity were observed due to the blade passage. It can be noted from Fig. 3e that a trailing vortex develops from the tip of the blade at an angle of 15° after the blade passage, detaching from the tip of the blade at *θ*=30° and decreasing in strength after a complete blade passage (*θ*=60°). The vortex originating from the passing blade is visible in Fig. 3e–h at a location *z*/*H*=0.11 and *r*/*R*=0.78, moving towards the bottom left-hand side as the blade progresses. The trailing tip vortex rotates in an anti-clockwise direction with *ω*_*θ*_/*N* values of up to 40 being measured. Above the impeller blade fluid is ejected outwards axially and radially, whilst beneath the impeller is an influx of liquid upwards towards the blade. The fluid ejected from the blade impinges on the bioreactor inner wall, with a portion of the fluid flowing downwards, thus inducing the counter-clockwise rotating fluid present in the lower quarter of the bioreactor. It is noteworthy that in the case of an air sparged bioreactor, the compartmentalisation of the fluid between the bottom quarter and upper three-quarters of the bioreactor would result in a larger gas residence time (Sardeing et al., 2004), with air bubbles becoming entrained within the lower ring vortex.

### 3.2 Variation of flow characteristics with *Re*

**Fig. 4:**
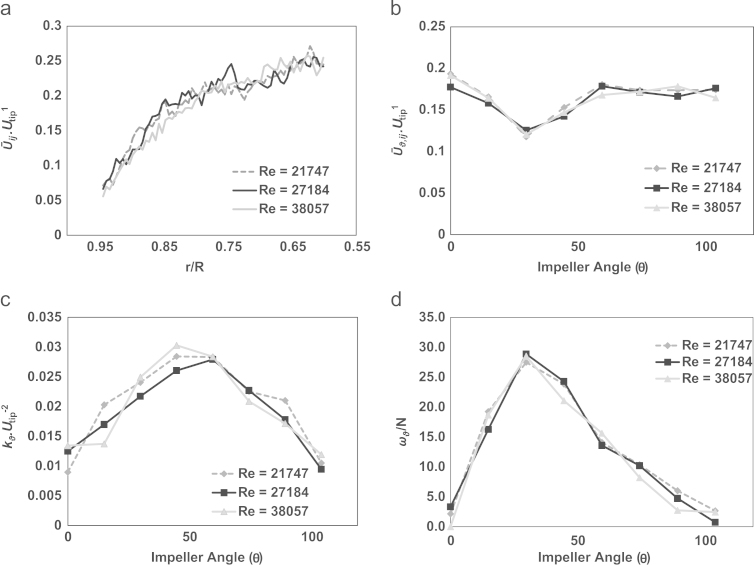
(a) Radial profile of the ensemble-averaged velocity magnitude at the impeller fluid discharge zone (*z*/*H*=0.148, *r*/*R*=0.381–0.944); (b) phase-resolved velocity at the impeller tip (*z*/*H*=0.128, *r/R*=0.652); (c) phase-resolved turbulent kinetic energy at the impeller tip (*z*/*H*=0.128, *r/R*=0.700); and (d) phase-resolved tangential vorticity at the impeller tip (*z*/*H*=0.128, *r/R*=0.652).

The velocity field data presented in Section 3.1 have helped elucidate the whole-field flow occurring in the 3 L CellReady bioreactor. It is now necessary to investigate in more detail the flow near the impeller where the greatest turbulence and stress levels are likely to occur. The radial profile of the ensemble-average velocity in correspondence of the impeller height and the variation of phase-resolved characteristics with impeller blade angle at different impeller speeds are presented in Fig. 4a–d. The radial ensemble-average velocity profile that extends from the impeller blade at radial positions *r*/*R*=0.381 to *r*/*R*=0.944 and at *z*/*H*=0.148 is presented in Fig. 4a. Velocity values change from a maximum of 0.25*U*_*tip*_ to 0.05*U*_*tip*_ at locations near the bioreactor wall, with no significant difference between results obtained at different Reynolds numbers. The phase-resolved velocity $\left({\overset{¯}{U}}_{\vartheta ,ij}/U_{tip}\right)$, turbulent kinetic energy $\left(k_{\vartheta }/U_{tip}^{2}\right)$ and tangential vorticity (*ω*_*θ*_/*N*) variation with blade angle are shown in Fig. 4b, c and d, respectively, at different impeller speeds and at axial and radial locations *z*/*H*=0.128 and *r*/*R*=0.652. The location selected is within the impeller exit stream close to the leading blade. In this work the isotropic assumption was used to determine the third *r*.m.s. fluctuating velocity component ($u_{k}^{\prime }$) and the ensemble-averaged and phase-resolved turbulent kinetic energy (*k* and *k*_*θ*_) were calculated from Eqs. (2) and (3):

(2) $$k=\frac{3}{4}(\overset{¯}{u_{i}^{2}}+\overset{¯}{u_{j}^{2}})$$

(3) $$k_{\vartheta }=\frac{3}{4}(\overset{¯}{u_{i}^{\prime \prime 2}}+\overset{¯}{u_{j}^{\prime \prime 2}})$$

The parameter u″ represents the turbulent velocity, with the periodic fluctuations due to the blade passage removed (Sharp and Adrian, 2001), and is calculated from phase-resolved measurements. From Fig. 4b it can be observed that the phase resolved velocity reaches a maximum of 0.2*U*_*tip*_ in correspondence to the blade passage (*θ*=0°), it decreases between *θ*=0° to 30° and increases again to a value of 0.18*U*_*tip*_ which then remains constant until the subsequent blade passage. Phase-resolved velocity and vorticity were found to scale linearly with *U*_*tip*_, whilst the turbulent kinetic energy scaled linearly with $U_{tip}^{2}$*,* in the impeller speed range considered, thus demonstrating that fully developed turbulent flow is present in the impeller region at the lowest Reynolds number at which measurements were obtained. Given the spatial variation of velocity, turbulent kinetic energy and vorticity, it can be concluded that a different location investigated would result in a different angular profile.

The flow characteristics within the impeller zone of the CellReady remain consistent at varying *Re* numbers. This is in agreement with the work of Bittorf and Kresta (2000), who showed that the active zone of mean circulation for an axial impeller is constant and does not depend on impeller diameter or impeller speed if these parameters are within the tested range (0.2*H*<*D*<0.6*H;* 13,500<*Re*<196,000). An increase in the impeller speed is only responsible for an increase in the fluid velocity magnitude. The location of the mean active impeller zone is dependent upon the impeller clearance (*C*) from the tank bottom, as well as the fluid discharge angle and the point at which the fluid impinges upon the wall (Bittorf and Kresta, 2000).

Multiphase flow measurements in the presence of the gas phase were not conducted in this work, however it is generally thought that the impeller speed will have an impact upon the spatial gas phase distribution. Visual observations of two-phase flow have suggested that significant flooding of the impeller is likely to occur in the CellReady bioreactor at impeller speeds below *N*=200 rpm (as noted by Kaiser et al., 2011. However, the type of impeller employed was found to have a significant impact upon the bubble breakage efficacy and gas flow pattern (Martín et al., 2008). The impact of a propeller on the gas size distribution within a stirred tank reactor was evaluated by Martín et al. (2008). In this work the propeller is observed deflecting (or redirecting) the bubbles rather than retaining them on the blade and breaking them. The bubble deformation, breakage and coalescence were shown to occur during entrainment of the gas phase beneath the propeller. The presence of two counter-rotating regions of flow in the CellReady suggests that entrainment of the gas phase may occur at impeller speeds higher than *N*=200 rpm, thus leading to a broader distribution of bubble size and a gradient of oxygen concentration across the height of the vessel.

**Fig. 5:**
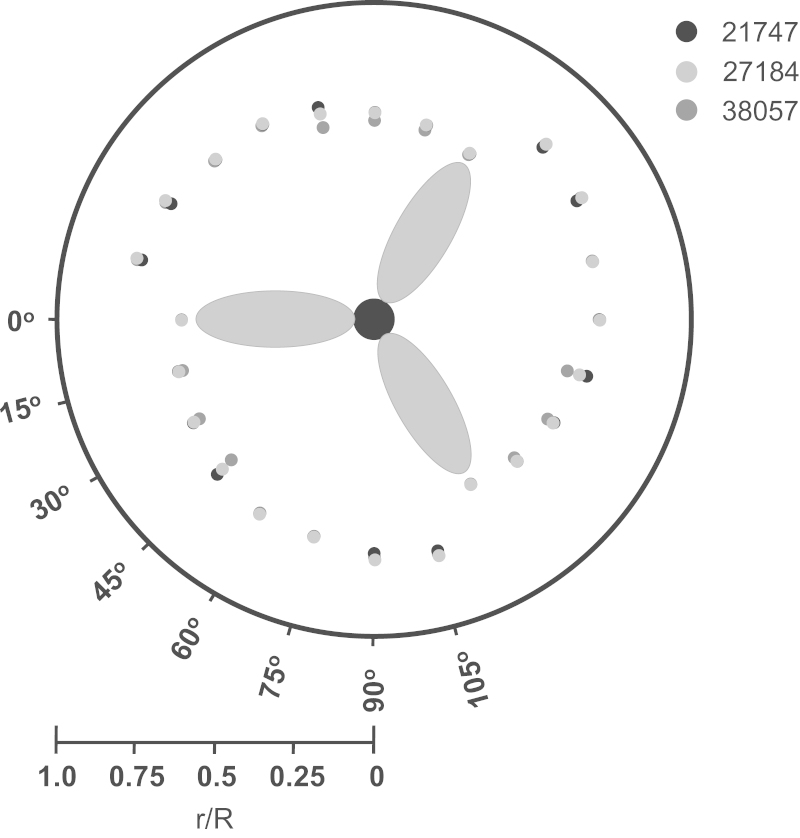
The radial location of the trailing tip vortex centre (top-down perspective) at *θ*=0°, 15°, 30°, 45°, 60°, 75°, 90°, and 105°, for *Re*=21,747, 27,184 and 38,057.

Fig. 5 displays the locations of the trailing tip vortex centre at impeller rates of 200, 250 and 350 rpm (*Re*=21,747, 27,184 and 38,057) and impeller angle increments of 15°. The radial location of the blade tip vortex was determined by identifying the position where the maximum vorticity occurs (located in the centre of the vortex, as determined by Schaefer et al. (1998)). The radial movement of the vortex away from the impeller tip (up to a distance of 0.10*T* from the impeller tip) is in agreement with the work of Khopkar et al. (2004), where a Rushton turbine (*D*=*T*/2) was studied using PIV. The results obtained in this work are, however, slightly in contrast with the trailing vortex profile observed by Schaefer et al. (1998) whereby a down-pumping 45° pitched blade turbine was investigated in a cylindrical vessel (*D*=0.329*T*) using LDA. In Schaefer et al. (1998), the vortex was noted moving radially by less than 0.0015*T*, along with a 20° downward inclination relative to the horizontal plane. The path of the trailing tip vortex and its wake does not change significantly as the impeller rate is increased from *N*=200 to 350 rpm. The maximum vorticity within the impeller zone is associated with the trailing tip vortex. Given the relatively high impeller to tank diameter ratio present in the CellReady bioreactor, the radial path travelled by the vortex is reasonably small, thus the impact of an increase or decrease in *Re* upon vortex radial trajectory is not significant.

The Reynolds stress field is defined as the apparent stress that results from turbulent velocity fluctuations (Munson et al., 2002) and it can be obtained from Eq. (4).

(4) $$-\rho \overset{¯}{u_{i}u_{j}}$$

**Fig. 6:**
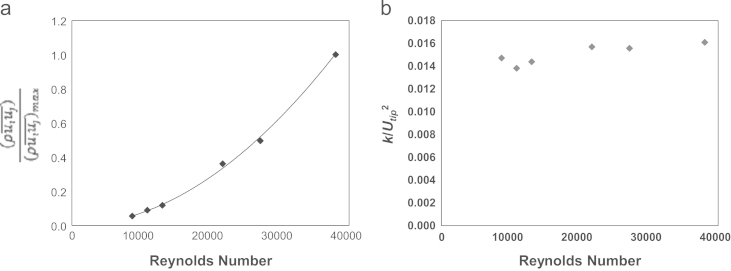
(a) Reynolds Stress and (b) turbulent kinetic energy in a selected impeller zone.

Fig. 6a and b presents the variation of the dimensionless ensemble-averaged Reynolds stress $(\rho \overset{¯}{u_{i}u_{j}})/{\left(\rho \overset{¯}{u_{i}u_{j}}\right)}_{max}$ and turbulent kinetic energy within the impeller zone as the impeller rate is increased from 80 to 350 rpm (*Re*=8699–38057). Data in Fig. 6 was obtained by taking the ensemble-average of the velocity values obtained at 8 different impeller blade angles. Thus each data point represents the average of 2000 vector maps. In addition, each data point in Fig. 6 represents the average of velocity values obtained in the fluid discharge region comprised between *r*/*R*=0.57 and 0.77 and *z*/*H*=0.11–0.13. As observed in Fig. 4b and d, phase resolved velocities and vorticity values were found to vary linearly, with *U*_*tip*_ and *N* respectively, for the range of Reynolds numbers investigated and no significant variation with *Re* was noted. In contrast, the variation of ensemble-averaged Reynolds stress with *Re* was found to be exponential. The Reynolds stress can be calculated from the product of the fluctuating components of the instantaneous velocity field, thus the level of Reynolds stress in the bioreactor increases significantly as the impeller rotational speed increases. Values up to 2.5 Pa at 350 rpm were measured. Such values are greater than the 0.4 Pa estimated for a 3.5 L BIOSTAT^®^ B-DCU STR (*D*/*T*=0.57) housing a 3-bladed pitched “elephant ear” impeller at *N*=150 rpm, *Re*=26,159 (Sorg et al., 2011). Shear stress values measured in this work are lower than the shear stress limit of 150 Pa, identified by Godoy-Silva et al. (2009) above which a fatal response by GS-CHO cells to the hydrodynamic stress is triggered, and lower than the shear stress limit (approximately 6 Pa) resulting in a change in recombinant protein glycosylation profile (Godoy-Silva et al., 2009). Indicating that the maximum stress levels measured in the CellReady, are not substantial enough to cause significant cell death or reduced cellular growth.

It is noteworthy that a maximum value of *k*_*θ*_ up to 0.03$U_{tip}^{2}$ was found from phase-resolved data in the impeller exit stream (Fig. 4c). The ensemble-averaged *k*, calculated in the impeller zone region between *r*/*R*=0.57 and 0.77 and *z*/*H*=0.11 to 0.13, remained constant at a value of 0.015–0.016 $U_{tip}^{2}$ at varying *Re* (Fig.6b). The maximum *k* values obtained in this work are greater than the maximum *k* of 0.02$U_{tip}^{2}$ observed by Aubin et al. (2004) for an axial up-pumping 45° pitched blade turbine. Gabriele et al. (2009) measured turbulent kinetic energy values near a 45° pitched blade turbine and found a maximum *k=*0.071$U_{tip}^{2}$ at a similar distance from the impeller tip (approximately 0.12*T*) than that observed in this work (approximately 0.10*T*).

The rate of viscous dissipation of the turbulent kinetic energy was determined from calculation of the spatial fluctuating velocity gradients as defined by Hinze (1975), using Eq. (5).

(5) $$\varepsilon =\nu \left[\begin{array}{c} 2\left(\overset{¯}{{\left(\frac{\partial u_{i}}{\partial x_{i}}\right)}^{2}}\;+\;\overset{¯}{{\left(\frac{\partial u_{j}}{\partial x_{j}}\right)}^{2}}\;+\;\overset{¯}{{\left(\frac{\partial u_{k}}{\partial x_{k}}\right)}^{2}}\;\right)+\overset{¯}{{\left(\frac{\partial u_{i}}{\partial x_{j}}\right)}^{2}}\;\\ +\;\overset{¯}{{\left(\frac{\partial u_{j}}{\partial x_{i}}\right)}^{2}}\;+\;\overset{¯}{{\left(\frac{\partial u_{i}}{\partial x_{k}}\right)}^{2}}\;+\;\overset{¯}{{\left(\frac{\partial u_{k}}{\partial x_{i}}\right)}^{2}\;\;}+\;\overset{¯}{{\left(\frac{\partial u_{j}}{\partial x_{k}}\right)}^{2}}\\ +\;\overset{¯}{{\left(\frac{\partial u_{k}}{\partial x_{j}}\right)}^{2}}+\;2\left(\overset{¯}{\frac{\partial u_{i}}{\partial x_{j}}\frac{\partial u_{j}}{\partial x_{i}}}+\;\overset{¯}{\frac{\partial u_{i}}{\partial x_{k}}\frac{\partial u_{k}}{\partial x_{i}}}+\;\overset{¯}{\frac{\partial u_{j}}{\partial x_{k}}\frac{\partial u_{k}}{\partial x_{j}}}\right) \end{array}\right]$$

The third component of the velocity was not measured in this work, hence Eq. (5) was simplified under the assumption of statistical isotropy. The seven remaining components were calculated using Eqs. (6)–(8) (Baldi et al., 2002).

(6) $$\overset{¯}{{\left(\frac{\partial u_{k}}{\partial x_{k}}\right)}^{2}}=\frac{1}{2}\left[\overset{¯}{{\left(\frac{\partial u_{i}}{\partial x_{i}}\right)}^{2}}\;+\;\;\overset{¯}{{\left(\frac{\partial u_{j}}{\partial x_{j}}\right)}^{2}}\right]\;$$

(7) $$\overset{¯}{{\left(\frac{\partial u_{i}}{\partial x_{k}}\right)}^{2}}=\overset{¯}{{\left(\frac{\partial u_{k}}{\partial x_{i}}\right)}^{2}}=\overset{¯}{{\left(\frac{\partial u_{j}}{\partial x_{k}}\right)}^{2}}=\overset{¯}{{\left(\frac{\partial u_{k}}{\partial x_{j}}\right)}^{2}}\;=\;\frac{1}{2}\left[\overset{¯}{{\left(\frac{\partial u_{i}}{\partial x_{j}}\right)}^{2}}+\;\overset{¯}{{\left(\frac{\partial u_{j}}{\partial x_{i}}\right)}^{2}}\right]$$

(8) $$\overset{¯}{\frac{\partial u_{i}}{\partial x_{k}}\frac{\partial u_{k}}{\partial x_{i}}}=\;\overset{¯}{\frac{\partial u_{j}}{\partial x_{k}}\frac{\partial u_{k}}{\partial x_{j}}}=\frac{-1/2\overset{¯}{{\left(\partial u_{i}/\partial x_{i}\right)}^{2}}+(-1/2\overset{¯}{{\left(\partial u_{j}/\partial x_{j}\right)}^{2}})}{2}=-\frac{1}{4}\left[\overset{¯}{{\left(\frac{\partial u_{i}}{\partial x_{i}}\right)}^{2}}+\overset{¯}{{\left(\frac{\partial u_{j}}{\partial x_{j}}\right)}^{2}}\right]$$

By substituting Eqs. (6)–(8) into (5), (9) was obtained and was used to estimate the energy dissipation rate of the turbulent kinetic energy.

(9) $$\varepsilon =v\;\left\{2\overset{¯}{{\left(\frac{\partial u_{i}}{\partial x_{i}}\right)}^{2}}\;+\;2\overset{¯}{{\left(\frac{\partial u_{j}}{\partial x_{j}}\right)}^{2}}\;+\;3\overset{¯}{{\left(\frac{\partial u_{i}}{\partial x_{j}}\right)}^{2}}\;+\;3\overset{¯}{{\left(\frac{\partial u_{j}}{\partial x_{i}}\right)}^{2}}+\;2\overset{¯}{\frac{\partial u_{i}}{\partial x_{j}}\frac{\partial u_{j}}{\partial x_{i}}}\right\}$$

**Fig. 7:**
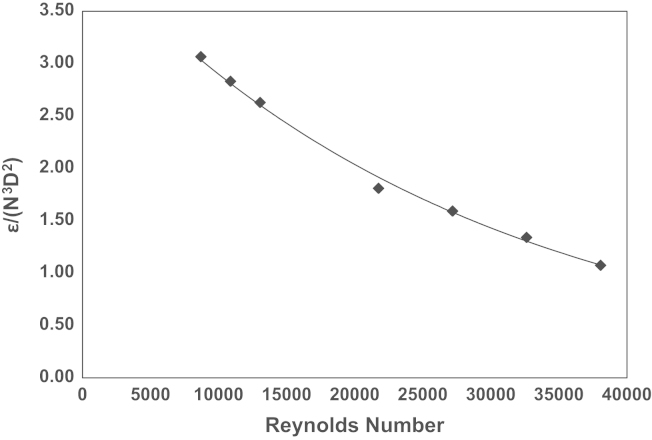
Ensemble-averaged *ε*/*N*^3^*D*^2^ within the impeller exit zone (*r*/*R*=0.54–0.79; *z*/*H*=0.13–0.17).

Fig. 7 shows the variation of the normalised energy dissipation rate (EDR), *ε*/*N*^3^*D*^2^, with *Re* obtained from time-resolved data (within the region *r*/*R*=0.54–0.79 and *z*/*H*=0.13–0.17). As expected the rate of energy dissipation (*ε*/*N*^3^*D*^2^) decreases as the Reynolds number is increased. Table 3 shows maximum energy dissipation rate data from the previous works of Baldi and Yianneskis (2004) and Zhou and Kresta (1996). Baldi and Yianneskis (2004) showed that *ε*_*max*_/*N*^3^*D*^2^ reach values of approximately 11 at the lower Reynolds numbers investigated (15,000–20000), whilst Zhou and Kresta (1996) note values below 3 at Reynolds numbers greater than 37,500. The accuracy of the energy dissipation rate measurement depends on the spatial resolution of the vector fields obtained, thus as the energy dissipation rate increases, the proportion of the actual energy dissipation rate that is measured will decrease (Baldi et al., 2002). Baldi and Yianneskis (2004) used the spatial fluctuating velocity gradients method (Hinze, 1975) to estimate the energy dissipation rate (as is the method used in this study), whereas Zhou and Kresta (1996) used the dimensional method. Therefore, obtaining a method that can estimate the EDR to as great an accuracy as possible, will be imperative to enhance comparability between different mixing systems.

### 3.3 Variation of flow characteristics with working volume

**Fig. 8:**
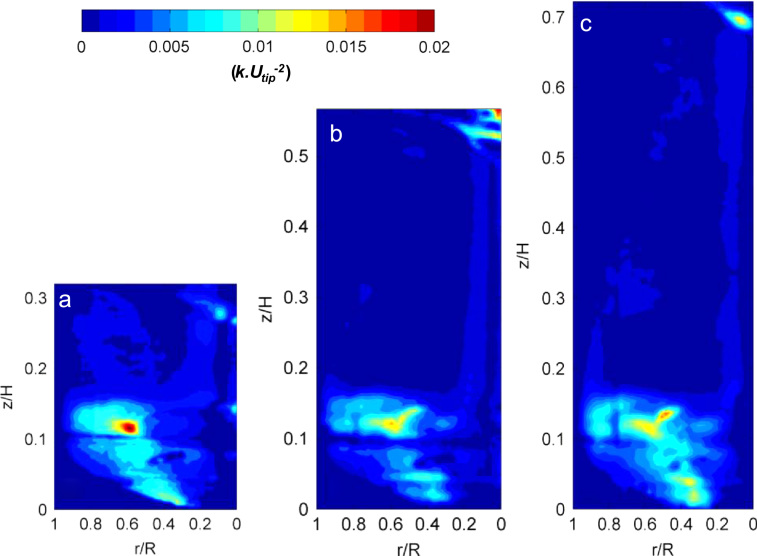
Ensemble-averaged turbulent kinetic energy (*k*) at *N*=200 rpm and fill volumes (a) 1.0 L (b) 1.8 L; (c) 2.4 L.

The results presented in Sections 3.1 and 3.2 have clearly elucidated the liquid flow pattern produced by the marine scoping impeller within the CellReady bioreactor and have indicated a fully developed turbulent flow is present, at least in the impeller zone, at the impeller speeds investigated in this work. The marine scoping impeller was found to produce a radial discharge flow and a lower circulation loop characterised by high average velocities (up to 0.36*U*_*tip*_ in phase-resolved measurements) and facilitated by the curved bottom shape of the bioreactor. The mean flow pattern in the CellReady was found to be similar to the radial flow pattern of a Rushton turbine (Schaefer et al., 1997) rather than to the flow produced by typical axial up-pumping impellers (Aubin et al., 2004; Gabriele et al., 2009),

Time-averaged velocity measurements were carried out at three different fill volumes and at a constant impeller speed of *N*=200 rpm (*Re*=21,747). Liquid fill volume is a parameter which has rarely been investigated with respect to its impact on fluid dynamics properties, however volume changes are typical within mammalian fed-batch cell culture operation due to sample removal and feed and base additions. Using Eq. 2, the ensemble-averaged turbulent kinetic energy was calculated from the turbulent fluctuations and results are shown in Fig. 8. At the lowest fill volume investigated in this work, an area of high energy is present at the bottom of the tank up to a height of *z*/*H*=0.15 where the bioreactor appears to be well mixed. However, as the fill volume is increased, the size of this area remains unchanged and therefore the region characterised by lower energy values, extending from *z*/*H*=0.2 to the liquid surface, represents a higher percentage of the total liquid volume present in the bioreactor. In these regions, corresponding to approximately the upper 75% of the bioreactor fill volume when 2.4 L volume was used, the maximum turbulent kinetic energy is less than 10% of the maximum *k* measured in the whole bioreactor. As it can be observed from Fig. 8, the region close to the impeller is characterised by maximum *k* values, rapidly decreasing in both radial and axial directions when moving away from the impeller tip location. The radial pumping motion induced by the impeller is evident in Fig. 8 where the maximum turbulent kinetic energy value decreases from 0.02 to 0.005$U_{tip}^{2}$ in the radial direction as the momentum is transferred outwards following the blade passage. The observed distribution of *k* both within the impeller zone and in the bulk of the bioreactor at the fill volumes investigated in this work may have implications for mass transfer and homogeneity during mammalian cell culture. Comparison with measurements obtained in similar stirred tank configurations (Gabriele et al., 2009) shows that the CellReady is characterised by lower levels of turbulence, likely due to the impeller clearance.

**Fig. 9:**
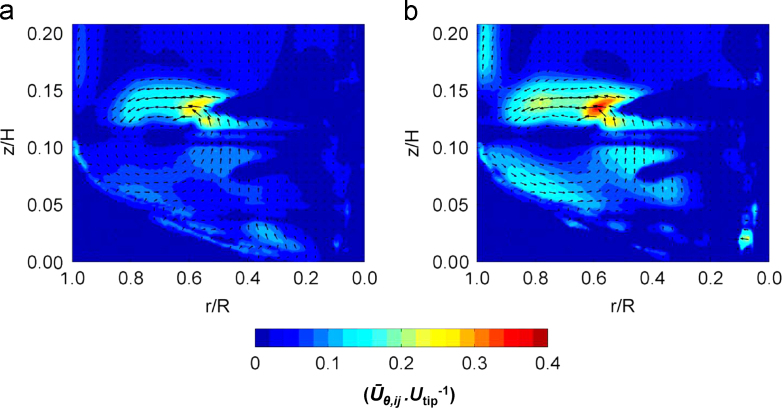
Phase-resolved velocity magnitude contour plot with vectors superimposed in the impeller zone at an angle *θ*=15° relative to blade: (a) 1.0 L and (b) 2.4 L fill volume.

**Fig. 10:**
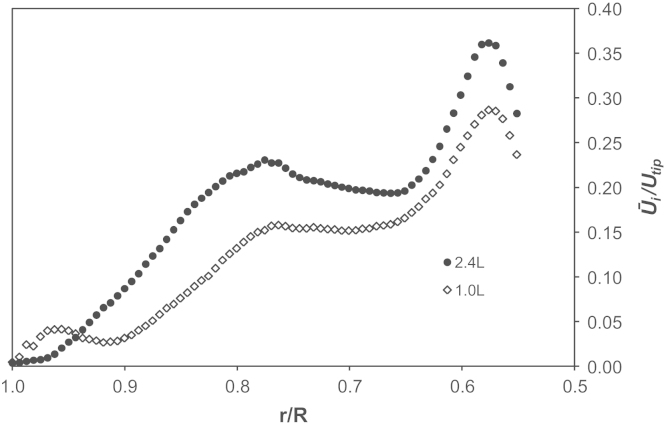
Radial profile of the ensemble-averaged radial velocity at *z*/*H*=0.09 at two different fill volumes.

Fig. 9 shows phase-resolved velocity magnitude data obtained at 1 and 2.4 L working volumes in an area of the bioreactor below *z*/*H*=0.2. A comparison between results plotted in Fig. 9a with those in Fig. 9b shows a significant difference in the fluid velocity in the impeller zone at the minimum and maximum fill volumes investigated. These plots are based on phase-resolved data at 15° azimuthal angle relative to the impeller blade. This angle was selected to show both the formation of the trailing vortex and the remainder of the vortex from the previous blade passage through the measurement area. It can be noted from Fig. 9b that the fluid in the trailing vortex reaches 0.36*U*_*tip*_, while at 1.0 L the maximum velocity is 0.29*U*_*tip*_. Fig. 10 shows a radial profile of the ensemble-averaged radial velocity from the impeller tip to the edge of the vessel at height *z*/*H*=0.09, positioned to intersect both vortices. The velocity reaches a maximum, for both volumes investigated, at *r*/*R*=0.58 in correspondence of a newly formed trailing vortex, while the peaks at *r*/*R*=0.76–0.78 represent the previous trailing vortex moving away from the impeller in the tangential direction. It can be observed from Fig. 9 that the velocity within both trailing vortices is greater at the greater fill volume, but also that the velocity of the trailing vortex is sustained for longer/further at the greater fill volume. Vortex direction, as with general flow, is directed more radially in comparison to previous studies where axial impellers were used, albeit down pumping (Khan et al., 2006; Schaefer et al., 1998). Finally, the maximum ensemble-averaged axial velocity observed in Fig. 10 at 0.97*r*/*R* at 1.0 L is probably in correspondence with the fluid impinging on the wall and moving upwards. This also occurs when a working volume of 2.4 L is used, however it occurs in a region above the measurement plane selected in Fig. 10. It can therefore be speculated that this compression of the flow pattern at 1.0 L fill volume conditions could account for the lower ensemble-averaged fluid velocity magnitude measured in the impeller zone.

**Fig. 11:**
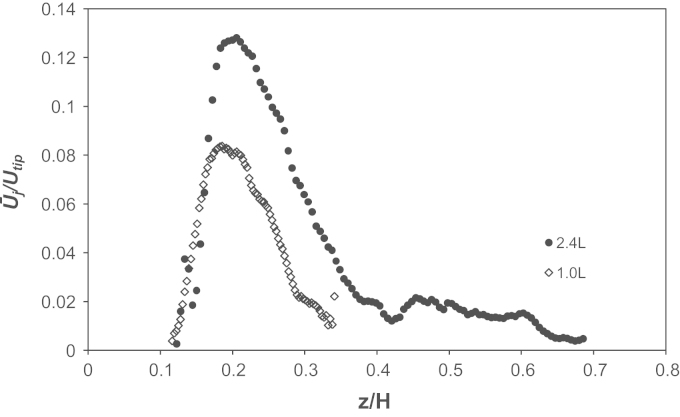
Axial profile of the ensemble-averaged axial velocity at *r*/*R*=0.95 at two different fill volumes.

Fig. 11 shows the axial profile of the ensemble-averaged axial velocity at *r*/*R*=0.95. In the figure it can be observed that for both fill volumes there is an axial flow moving up the side of the vessel generated from the impeller discharge stream impinging on the vessel wall. A jet of fluid moves vertically upwards in a region close to the wall at 0.9–1.0*r*/*R*, with maximum values of 0.8 and 0.13*U*_*tip*_ at 1.0 and 2.4 L, respectively, observed at a radial location of *r*/*R*=0.97. At the higher fill volume investigated in this work, the liquid height, *H*_*L*_, is greater and the fluid therefore has a longer time over which to decelerate before reaching the liquid surface. This can be seen in the longer trace and higher velocities measured (peak 0.13*U*_*tip*_). At a fill volume of 1 L the liquid surface impinges on the flow up the side before it has fully decelerated and this is likely to cause the wall jet to slow down with peak 0.083*U*_*tip*_ but also force this flow slightly down the vessel. The lower velocity values observed within the wall jet may be responsible for the lower velocities observed within the impeller zone and presented in Fig. 9.

### 3.4 Impact of hydrodynamic conditions on cell culture

**Fig. 12:**
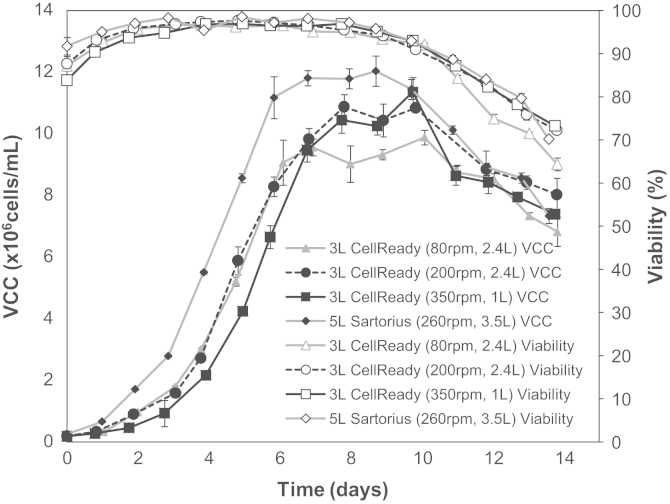
Viable cell count (×10^6^ cells/mL) and viability (%) profiles for the CellReady fed-batch cell cultures conducted at 200 rpm—2.4 L, 350 rpm—1 L and 80 rpm—2.4 L conditions and the Sartorius fed-batch cell culture conducted at 260 rpm—3.5 L.

The results obtained using the PIV system presented in Sections 3.1–3.3 have helped elucidate some of the flow characteristics in the 3 L Mobius CellReady and their variation with bioreactor operating conditions. In order to ascertain the performance of an antibody-producing CHO cell-line within the CellReady in response to a change in hydrodynamic conditions, cell culture experiments were conducted in the same bioreactor. For each experiment cellular growth, protein productivity and metabolite production of fed-batch cell cultures at the conditions reported in Table 2 were obtained. The fluid working volume and impeller rotation rates selected for the cell culture investigation are within the Millipore recommended range of fluid working volume and impeller rate operating parameters. Three impeller speed-liquid fill volume combinations were selected to represent the upper (350 rpm and 1 L), middle (200 rpm and 2.4 L) and lower (80 rpm and 2.4 L) levels of turbulence occurring within the CellReady. The reduction of fluid working volume from 2.4 L to 1 L would serve to increase the frequency with which cells pass through the lower circulation loop characterised by higher turbulence levels and higher Reynolds stresses. For simplicity, these three experimental conditions will be referred to as 80 rpm—1 L, 200 rpm—2.4 L and 350 rpm—1 L. Fig. 12 shows the viable cell growth profiles and cell viability at the three cell culture conditions tested. Cell growth and viability data of a benchmark cell culture conducted in a 5 L Sartorius bioreactor is also reported in Fig. 12. For most operating conditions and bioreactor systems viable cell density reached values higher than 10×10^6^ cells/mL. The growth profile obtained from the cell culture conducted at 350 rpm and 1 L volume is characterised by a longer lag phase than the profiles obtained at the other conditions. An extended lag phase usually indicates a period of adaptation and in this case might be an indication that cells are adapting to the higher turbulence levels present in the bioreactor at the 350 rpm—1 L condition. Under these conditions cells achieve a maximum concentration of over 11×10^6^ cells/mL, thus showing a similar profile to the other cultures in the exponential and stationary phases. This is in agreement with the work of Kunas and Papoutsakis (1990), whereby even in the presence of entrained bubbles (ranging from 50 to 300 µm) and impeller speeds of up to *N*=700 rpm, good cell growth of hybridoma cultures was observed in a 2 L Setric Genie Bioreactor.

Particle size distribution was measured using a sample at the end of each cell culture experiment to determine whether the selection of the higher impeller speed and lower liquid volume combination had an impact on cell size. Interestingly, particle size experiments showed a significantly reduced maximum cell diameter of 16.23 µm was obtained at 350 rpm—1 L conditions, whilst at 80 rpm—2.4 L and 200 rpm—2.4 L maximum cell diameters of 17.99 µm and 18.46 µm respectively were recorded (results not shown). Godoy-Silva et al. (2009) have shown that cell size during culture is influenced by repetitive cycles of high hydrodynamic stress levels. In the aforementioned study, cells were cyclically subjected to a “torture chamber” with energy dissipation rate value of 6.4×10^6^ W/m^3^, from day 4 of 14 days-long CHO cell culture. The cell culture experiments were conducted within a 2 L (1 L working volume) bioreactor with dual pitched blade impellers (Applikon, Inc., Foster city, CA) at *N*=130 rpm. Cells not subjected to the “torture chamber” attained a final day mean cell diameter of approximately 18 µm, whilst those exposed to the repetitive increased energy dissipation rates were found to have a mean cell diameter of 17 µm. In addition to the influence of the rate of energy dissipation on cell size during culture, this finding is important in relation to the flow length scale to cell size ratio. It has been suggested that if the Kolmogorov length scale is greater than the cell diameter, then cell damage should not occur (Scott et al., 2012), since cells would become entrained within the larger turbulent eddies rather than collide with turbulent eddies of size comparable to theirs.

**Fig. 13:**
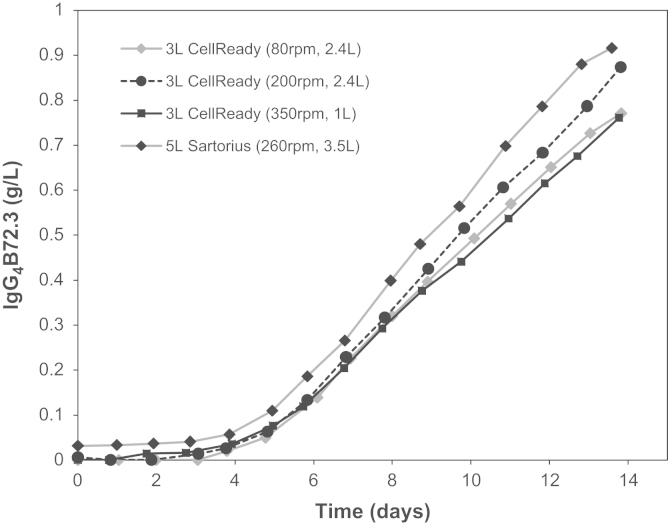
IgG_4_ B72.3 (g/L) profiles for the CellReady fed-batch cell cultures conducted at 200 rpm—2.4 L, 350 rpm—1 L and 80 rpm—2.4 L conditions and the Sartorius fed-batch cell culture conducted at 260 rpm—3.5 L.

Fig. 13 shows the IgG_4_ concentration present in samples obtained daily throughout the duration of the 14 day-long cultures. The maximum recombinant protein concentration of 0.92 g/L was expressed by cells grown in the 5 L Sartorius bioreactor, while those cultivated in the CellReady at *N*=200 rpm and *V*_*L*_=2.4 L obtained an IgG_4_ titre of 0.87 g/L. Cell culture experiments conducted at 80 rpm—2.4 L and 350 rpm—1 L resulted in lower recombinant protein production of 0.77 g/L and 0.76 g/L respectively (a reduction of 17% and 18% respectively in comparison to the benchmark Sartorius experiment). Cells grown at the 80 rpm—2.4 L condition did not attain the same maximum cell density as their counterparts (200 rpm—2.4 L and 350 rpm—1 L). In the case of cells grown at *N*=80 rpm it is possible that nutrient and oxygen limitations occurred due to insufficient mixing. As a result of the reduced *Re*, the cells began to form clusters which would have impacted upon nutrients transport to cells. The latter observation is based on visual inspection of the images taken of cells. The lower viable cell count observed at 80 rpm—2.4 L and the lower cell specific productivity found at this condition is likely to have influenced the final IgG_4_ titre. Based on the fluid dynamic investigations, *Re=*21,747 corresponds to the onset of fully developed turbulent flow conditions along with gas phase entrainment, but it is likely that cells in regions above the impeller experienced significantly reduced mass transfer conditions. This disparity in fluid dynamic stress is even more pronounced between the two counter-rotating loops observed at 350 rpm. The reduced IgG_4_ titre observed at 350 rpm—1 L is in agreement with the work of Nienow et al. (2013b), where CHO cells grown within a 2 L STR were repeatedly subjected to a plug flow loop with increased specific power input of up to 2.9×10^5^ W/m^3^. The cells were circulated through the loop at a similar frequency with which they would circulate as a result of agitation at the large scale. Cells grown within the STR with and without the recirculation loop obtained cell densities within batch to batch experimental deviation, however, cells that were repeatedly exposed to increased specific power input displayed a 20% reduction in IgG_4_ titre and cell specific protein productivity.

**Fig. 14:**
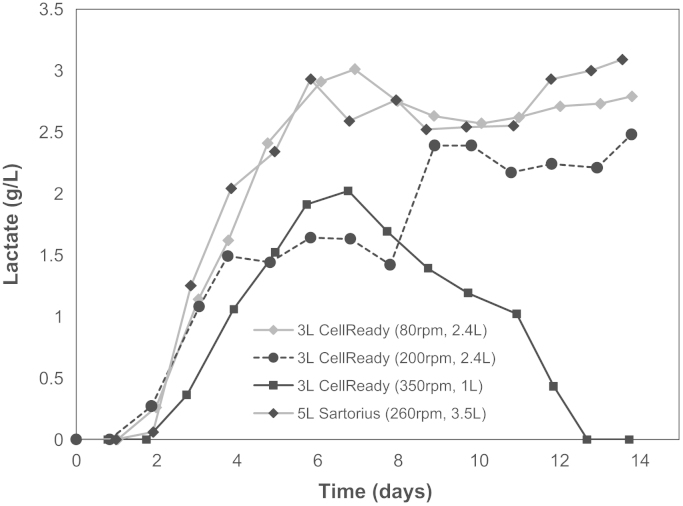
Lactate concentration profiles for the CellReady cell cultures conducted at 80 rpm—2.4 L, 200 rpm—2.4 L and 350 rpm—1 L conditions, as well as the 5 L Sartorius cell culture conducted at 260 rpm—3.5 L.

Profiles of metabolite concentrations (as described in Section 2.4) were obtained for all cell culture runs. Fig. 14 shows the lactic acid concentration profile for the three CellReady cell cultures conducted in this work. The lactate concentration reached the highest values of 3 g/L at 80 rpm—2.4 L. This correlates with the work of Sorg et al. (2011) which shows greater lactate production was obtained as the energy dissipation rate range becomes narrower. This highlights the importance of spatial and temporal homogeneity not only with respect to oxygen and nutrients concentration but also with regards to energy dissipation. The lactic acid concentration profile observed at 200 rpm—2.4 L is the profile expected for the CHO cell line used in this work, where the lactic acid increases upon commencement of the cell exponential growth phase. This behaviour is associated with the metabolism of glucose through glycolysis, followed by the conversion of pyruvate to lactate due to insufficient oxygen during the exponential phase (Campbell and Reece, 2005). Subsequently, as the stationary phase progresses, lactate concentration becomes constant (indicating net lactate consumption) due to the cellular demand for a higher carbon source concentration. In the case of the cells grown at 350 rpm—1 L, lactic acid concentration reaches a maximum at day 7 (which coincides with the beginning of the stationary phase) and then decreases for the remainder of the culture. At this stage lactate is metabolised in the Krebs cycle, which may indicate one of two scenarios: an increase in the demand for pyruvate through the Kreb׳s cycle, or reduced production of the pyruvate through glycolysis due to the sequestering of glucose within another metabolic pathway. The latter would result in a reduction in NADH and ATP production necessary for protein synthesis.

Lactate consumption is typically a cellular response to a lack of glucose as a carbon source, however, adequate glucose levels (>2 g/L) were maintained throughout the fed-batch cultures. The consumption of both glucose and lactate along with reduced IgG_4_ productivity may be an indication that the higher turbulence levels at 350 rpm—1 L engendered the reallocation of glucose away from IgG_4_ synthesis, towards more essential requirements. Table 4 shows cell specific productivity obtained during the stationary phase at the different conditions tested. The cell specific protein productivity at 350 rpm—1 L was 12% lower than the productivity obtained at 200 rpm—2.4 L, whilst the 80 rpm—2.4 L condition and the experiments conducted using the Sartorius bioreactor had cell specific productivities similar to that obtained at 200 rpm—2.4 L condition. This finding supports the aforementioned hypothesis about a possible change in glucose metabolism during the stationary phase. It can be postulated that the reason behind the shift from lactate production to consumption is oxidative stress. Oxidative stress is defined as a state of inequity within a cell where the reactive oxidative species becomes imbalanced in favour of the oxidant species. The adoption of high impeller rates and airflow inlet in the bioreactor may have resulted in locally high levels of oxygen leading to the oxidative stress phenomena (Mckenna, 2009). For this reason cells may have reduced their utilisation of glucose for protein production in order to maintain a reduced environment. Metabolic analysis of CHO cells has shown a stationary phase characterised by a reduced flux of glycolysis, net lactate uptake accompanied by significant glucose flux through the oxidative pentose phosphate pathway (oxPPP) (Ahn and Antoniewicz, 2011). This is in contrast to the exponential phase, where glycolysis contributes more (in comparison to the stationary phase) to ATP production (Ahn and Antoniewicz, 2011). The augmented oxPPP flux indicates a requirement of additional NADPH at the stationary phase to be used to counteract oxidative stress (Sengupta et al., 2011). Cells possess defensive enzymes such as glutathione peroxidise; these enzymes eliminate peroxides that accumulate within the cell, by oxidising glutathione to glutathione disulphide. This is subsequently regenerated by NADPH reduction catalysed by glutathione reductase (Sengupta et al., 2011).

## 4 Conclusion

In this work, particle image velocimetry has been used to obtain whole-field velocity characteristics within a commercially available single-use bioreactor. Cell cultures have been performed within the 3 L Mobius CellReady at 80 rpm—2.4 L, 200 rpm—2.4 L and 350 rpm—1 L, with the intention of varying the fluid dynamic environment to which the cells are exposed and to examine the impact of these conditions upon cell culture performance. The investigation of hydrodynamic parameters and their impact upon cells has indeed been conducted in recent literature, however, such studies have been mainly limited to ascertaining tolerance levels for parameters such as the viscous dissipation of the turbulent kinetic energy. The whole-field quantification of hydrodynamic parameters within a bioreactor using PIV allows a more detailed and rigorous study of the local conditions cells experience.

Given the distinct shifts in metabolic behaviour and the changes in productivity and cellular growth observed, it can be concluded that the fluid dynamic conditions can be used to impact upon cell performance. The compartmentalised regions of turbulence present throughout the bioreactor and identified using PIV, allows one to better understand the localised environmental conditions cells experience during the culture. The range of turbulence and velocity levels measured at the different operating conditions, correlated with the disparate cellular metabolic responses, and the changes to cell physiology and recombinant protein productivity exhibited by the GS-CHO cells.

Although issues with large-scale facility fit, bag expenses, supply and the potential impact of SUBs on process and product quality have obstructed the production of larger scale SUBs (Krishnan and Chen, 2012), the problems associated with scaling based upon traditional parameters (e.g., *Re*, *k*_*L*_*a*, *U*_*tip*_ etc.) are still present (Mollet et al., 2007). These parameters represent the mean flow, whilst cells are responsive to their local environment. Although the data presented cannot be used in isolation for scaling procedures, being a rigid bioreactor offers an indication of the possible flow conditions within similar vessels (e.g., un-baffled, axial impeller bioreactors with comparable geometric ratios) at a larger scale. The aim for the future would be to elucidate the fluid dynamic parameters that are both pertinent to cell culture behaviour and applicable to the array of different mixing strategies available on the market. Acquisition of dimensionless parameters at the localised and whole-field level within the bioreactor presented, along with that of others is necessary, and is currently being conducted. This would engender greater efficacy in scale-up procedures, in addition to enhanced cross-compatibility between bioreactor types.

## Nomenclature

***Roman characters***
*C*: clearance of impeller from tank bottom, mm
*C*_*i*_: viable cell concentration on day *i* of cell culture, cells/mL
*D*: impeller diameter, mm
*T*: vessel internal diameter, mm
*H*: bioreactor height, mm
*H*_*L*_: liquid height, mm
*IVC*_*i*_: integral viable cell concentration from day 0 to *i* of cell culture, cells day/mL
k: turbulent kinetic energy from time-resolved measurements, m^2^ s^−2^
$k_{\vartheta }$: turbulent kinetic energy from phase-resolved measurements, m^2^ s^−2^
*N*: impeller rotational speed (revolutions per second), s^−1^
*R*: CellReady bioreactor internal radius, m
*r*: radial direction distance, m
*Re*: Reynolds number $\left(=\rho {\mathrm{ND}}^{2}/\mu \right)$, dimensionless
*t*_*i*_: elapsed time of cell culture on day *i*, hours
$U_{i},U_{j},U_{k}$: radial, axial and tangential components of instantaneous velocity, m s^−1^
${\overset{¯}{U}}_{i},{\overset{¯}{U}}_{j},{\overset{¯}{U}}_{k}$: ensemble-averaged radial, axial and tangential velocity, m s^−1^
$U_{tip}$: impeller tip speed (=πDN), m s^−1^
${\overset{¯}{U}}_{ij}$: ensemble-averaged magnitude of radial and axial velocity components, m s^−1^
${\overset{¯}{U}}_{\vartheta ,ij}$: phase-resolved magnitude of radial and axial velocity components at phase *θ*, m s^−1^
$u_{i},u_{j},u_{k}$: radial, axial and tangential fluctuating velocity components $\left(u_{i}=U_{i}-{\overset{¯}{U}}_{i}\right)$, m s^−1^
$u_{i}^{\prime },\;u_{j}^{\prime },u_{k}^{\prime }$: root-mean-square (r.m.s.) of the fluctuating velocity components $\left(u_{i}^{\prime }=\sqrt{\overset{¯}{{u_{i}}^{2}}}\right)$, m s^−1^
$u_{ij}^{\prime }$: root-mean-square (r.m.s.) of the fluctuating velocity components (in axial and radial direction) $\left(u_{ij}^{\prime }=\sqrt{\left(1/2)(\overset{¯}{u_{i}^{2}}+\overset{¯}{u_{j}^{2}}\right)}\right)$, m s^−1^
$u_{i}^{\prime \prime },\;u_{j}^{\prime \prime },u_{k}^{\prime \prime }$: radial, axial and tangential turbulent fluctuations $\left(u_{i}^{\prime \prime }=U_{i}-{\overset{¯}{U}}_{\vartheta ,i}\right)$, m s^−1^
*V*_*L*_: bioreactor liquid volume, L
$x_{i},x_{j},x_{k}$: radial, axial and tangential distance, m
*z*: axial direction distance, m

***Greek characters***
ε: rate of viscous dissipation of the turbulent kinetic energy, m^2^ s^−3^
*θ*: tangential direction and phase angle, deg
*μ*: dynamic viscosity, kg m^−1^ s^−1^
ν: kinematic viscosity, m^2^ s^−1^
*ρ*: fluid density, kg m^−3^
*ω*_*θ*_: Phase-resolved vorticity around the tangential axis, s^−1^

***Abbreviations***
2-D: two dimensional
CHO: Chinese hamster ovary
DOT: dissolved oxygen tension
EDR: energy dissipation rate
GS: glutamine synthetase
LDA: laser Doppler anemometry
MAb: monoclonal antibody
mM: millimolar
mOsm: milliosmoles
PIV: particle image velocimetry
pg: picograms
oxPPP: oxidative pentose phosphate pathway
RO: reverse osmosis
rpm: revolutions per minute
r.m.s.: root mean square
STR: stirred tank reactor
SUB: single-use bioreactor
vvm: volume of air per volume of culture per minute

## Figures and Tables

**Table 1:** Sartorius BIOSTAT^®^ B-DCU and Mobius^®^ CellReady vessel and impeller dimensions.

Bioreactor	Sartorius BIOSTAT^®^ B-DCU	Mobius^®^ CellReady
Vessel height (*H*)	350 mm	249 mm
Vessel diameter (*T*)	160 mm	137 mm
Impeller diameter (*D*)	0.44*T*	0.56*T*
Blade clearance (*C*)	0.50*T*	0.22*T*

**Table 2:** Operating conditions used for the cell culture experiments in the CellReady bioreactor.

Operating Conditions			
Working volume (L)	1	2.4	2.4
Impeller rate (rpm)	350	200	80

**Table 3:** Variation of maximum normalised energy dissipation rate (*ε*_*max*_/*N*^3^*D*^2^) at varying *Re*.

	***Re***	***ε***_***max***_**/*****N***^**3**^***D***^**2**^		***Re***	***ε***_***max***_**/*****N***^**3**^***D***^**2**^		***Re***	***ε*****/*****N***^**3**^***D***^**2**^
Baldi and Yianneskis (2004**)**	15,000	11	Zhou and Kresta (1996**)**	37,500	2.47	**This study**	8699	3.07
	20,000	11		50,400	2.36		10,873	2.83
	25,000	9	Pitched blade turbine	60,900	2.62		13,048	2.63
Rushton turbine	32,000	10		73,600	2.33	Marine scoping impeller	21,747	1.81
	40,000	7		84,000	2.44		27,184	1.59
							32,620	1.34
							38,057	1.07

**Table 4:** Cell specific productivity of IgG_4_ (picograms per cell per day).

**Bioreactor & conditions**	**IgG**_**4**_**(pg/cell/day)**
5 L STR 260 rpm—5 L	9.3
CellReady 80 rpm—2.4 L	9.0
CellReady 200 rpm—2.4 L	9.3
CellReady 350 rpm—1 L	8.2

## References

[bib1] Abu-Reesh I., Kargi F. (1991). Biological responses of hybridoma cells to hydrodynamic shear in an agitated bioreactor. Enzyme Microb. Technol..

[bib2] Ahn W.S., Antoniewicz M.R. (2011). Metabolic flux analysis of CHO cells at growth and non-growth phases using isotopic tracers and mass spectrometry. Metab. Eng..

[bib3] Aubin J., Le Sauze N., Bertrand J., Fletcher D., Xuereb C. (2004). PIV measurements of flow in an aerated tank stirred by a down- and an up-pumping axial flow impeller. Exp. Therm. Fluid Sci..

[bib4] Baldi, S., Hann, D., Yianneskis, M., 2002. On the measurement of turbulence energy dissipation in stirred vessels with PIV techniques. In: Proceedings of the 11th International Symposium on Applied Laser Techniques in Fluid Mechanic. Lisbon. pp. 1–12.

[bib5] Baldi S., Yianneskis M. (2004). On the quantification of energy dissipation in the impeller stream of a stirred vessel from fluctuating velocity gradient measurements. Chem. Eng. Sci..

[bib6] Barbaroux M., Sette A. (2006). Properties of Materials Used in Single-Use Flexible Containers: Requirements and Analysis. Biopharm. Int..

[bib7] Business Insights, 2007. Biomanufacturing Strategies: Market Drivers, Build-vs.-Buy Decisions and Opportunities in Contract Relationship Management.

[bib8] Bittorf K.J., Kresta S.M. (2000). Active volume of mean circulation for stirred tanks agitated with axial impellers. Chem. Eng. Sci..

[bib9] Campbell N., Reece J. (2005). Biology.

[bib10] Cherry R.S. (1993). Animal cells in turbulent fluids: details of the physical stimulus and the biological response. Biotechnol. Adv.

[bib11] Chimica I., Bonino P.G.B., Pia O., Converti A., Zilli M., Arni S., Felice R. Di, Borghi M. Del (1999). Estimation of viscosity of highly viscous fermentation media containing one or more solutes. Biochem. Eng. J..

[bib12] Clincke M.F., Mölleryd C., Zhang Y., Lindskog E., Walsh K., Chotteau V. (2013). Very high density of CHO cells in perfusion by ATF or TFF in WAVE bioreactor^™^. Part I. Effect of the cell density on the process. Biotechnol. Prog..

[bib13] Deen N., Hjertager B. (2002). Particle image velocimetry measurements in an aerated stirred tank. Chem. Eng. Commun..

[bib14] Ducci a., Yianneskis M (2005). Direct determination of energy dissipation in stirred vessels with two-point LDA. AIChE J..

[bib15] Eibl R., Werner S., Eibl D. (2009). Bag bioreactor based on wave-induced motion: characteristics and applications. Adv. Biochem. Eng. Biotechnol..

[bib16] Gabriele A., Nienow A.W., Simmons M.J.H. (2009). Use of angle resolved PIV to estimate local specific energy dissipation rates for up- and down-pumping pitched blade agitators in a stirred tank. Chem. Eng. Sci..

[bib17] Godoy-Silva R., Chalmers J.J., Casnocha S.A., Bass L.A., Ma N. (2009). Physiological responses of CHO cells to repetitive hydrodynamic stress. Biotechnol. Bioeng..

[bib53] Wave [WWW Document], n.d. GE Healthc. Life Sci. 〈http://www.gelifesciences.com/webapp/wcs/stores/servlet/catalog/en/GELifeSciences-UK/brands/wave〉.

[bib18] Hill, D., Troolin, D., Walters, G., Lai, W., Sharp, K., 2008. Volumetric 3-component velocimetry (V3V) measurements of the turbulent flow in stirred tank reactors. In: Proceedings of the 14th International Symposium on Applications of Laser Techniques to Fluid Mechanics. pp. 1–12.

[bib19] Hinze J.O. (1975). Turbulence.

[bib20] Kaiser S.C., Eibl R., Eibl D. (2011). Engineering characteristics of a single-use stirred bioreactor at bench-scale: the Mobius CellReady 3 L bioreactor as a case study. Eng. Life Sci..

[bib21] Khan F.R. (2005). Investigation of Turbulent Flows and Instabilities in a Stirred Vessel Using Particle Image Velocimetry.

[bib22] Khan F.R., Rielly C.D., Brown D.A.R. (2006). Angle-resolved stereo-PIV measurements close to a down-pumping pitched-blade turbine. Chem. Eng. Sci..

[bib23] Khopkar A., Aubin J., Rubio-Atoche C., Xuereb C., Le Sauze N., Bertrand J., Ranade V.V. (2004). Flow generated by radial flow impellers: PIV measurements and CFD simulations. Int. J. Chem. React. Eng..

[bib24] Kretzmer G., Schiigerl K. (1991). Response of mammalian cells to shear stress. Appl. Microbiol. Biotechnol..

[bib25] Krishnan R., Chen H. (2012). A comprehensive strategy to evaluate single-use bioreactors for pilot-scale cell culture production. Rev. Am. Pharm. Bus. Technol..

[bib26] Kunas K.T., Papoutsakis E.T. (1990). Damage mechanisms of suspended animal cells in agitated bioreactors with and without bubble entrainment. Biotechnol. Bioeng..

[bib27] Löffelholz C., Husemann U., Greller G., Meusel W., Kauling J., Ay P., Kraume M., Eibl R., Eibl D. (2013). Bioengineering parameters for single-use bioreactors: overview and evaluation of suitable methods. Chem. Ing. Tech..

[bib28] Ma N., Koelling K.W., Chalmers J.J. (2002). Fabrication and use of a transient contractional flow device to quantify the sensitivity of mammalian and insect cells to hydrodynamic forces. Biotechnol. Bioeng..

[bib29] Martín M., Montes F.J., Galán M.A. (2008). Bubbling process in stirred tank reactors I: agitator effect on bubble size, formation and rising. Chem. Eng. Sci..

[bib30] Mckenna T. (2009). Oxidative stress on mammalian cell cultures during recombinant protein expression.

[bib31] Meyers J., Sagaut P. (2006). On the model coefficients for the standard and the variational multi-scale Smagorinsky model. J. Fluid Mech..

[bib32] Mollet M., Godoy-Silva R., Berdugo C., Chalmers J.J. (2007). Acute hydrodynamic forces and apoptosis: a complex question. Biotechnol. Bioeng..

[bib33] Munson B.R., Young D.F., Okiishi T. (2002). Fundamentals of Fluid Mechanics.

[bib34] Nienow A.W., Rielly C.D., Brosnan K., Bargh N., Lee K., Coopman K., Hewitt C.J. (2013). The physical characterisation of a microscale parallel bioreactor platform with an industrial CHO cell line expressing an IgG4. Biochem. Eng. J..

[bib35] Nienow A.W., Scott W.H., Hewitt C.J., Thomas C.R., Lewis G., Amanullah A., Kiss R., Meier S.J. (2013). Scale-down studies for assessing the impact of different stress parameters on growth and product quality during animal cell culture. Chem. Eng. Sci. Des..

[bib36] Oh S., Nienow A., Alrubeai M., Emery A. (1989). The effects of agitation intensity with and without continuous sparging on the growth and antibody production of hybridoma cells. J. Biotechnol.

[bib37] Pan C., Min J., Liu X., Gao Z. (2008). Investigation of Fluid Flow in a Dual Rushton Impeller Stirred Tank Using Particle Image Velocimetry. Chinese J. Chem. Eng..

[bib38] Pavlou A.K., Reichert J.M. (2004). Recombinant protein therapeutics--success rates, market trends and values to 2010. Nat. Biotechnol..

[bib39] Petersen J.F., McIntire L.V., Papoutsakis E.T. (1988). Shear sensitivity of culture hybridoma cells (CRL-8018) depends on mode of growth, culture age and metabolite concentration. J. Biotechnol..

[bib40] Petersen J.F., McIntire L.V., Papoutsakis E.T. (1990). Shear Sensitivity of hybridoma cells in batch, fed-batch, and continuous cultures. Biotechnol. Prog..

[bib41] Plion, P., Costes, J., Couderc, J.P., 1985. Study by Laser Doppler Anemometry of the Flow Induced by a Propeller in a Stirred Tank – Influence of Baffles. In: Proceedings of the 5th European Conference on Mixing. Wurzburg, Germany, pp. 341–353.

[bib42] Reichert J.M., Rosensweig C.J., Faden L.B., Dewitz M.C. (2005). Monoclonal antibody successes in the clinic. Nat. Biotechnol..

[bib43] Sardeing R., Aubin J., Xuereb C. (2004). GAS–LIQUID MASS TRANSFER: a comparison of down- and up-pumping axial flow impellers with radial impellers. Chem. Eng. Res. Des..

[bib44] Schaefer M., Hofken M., Durst F. (1997). Detailed LDV measurements for visualization of the flow field within a stirred-tank reactor equipped with a rushton turbine. Chem. Eng. Sci. Des..

[bib45] Schaefer M., Yianneskis M., Wachter P., Durst F. (1998). Trailing vortices around a 45 degree pitched-blade impeller. AIChE J..

[bib46] Schmid G., Huber F., Kerschbaumer R., Spier R.E., Griffiths J.B., Macdonald C. (1992). Adaptation of hybridoma cells to hydrodynamic stress under continuous culture conditions.

[bib47] Scott, W.H., Thomas, C.R., Hewitt, C.J., Lewis, G., Meier, S.J., Amanullah, A., Kiss, R., Nienow, A.W., 2012. Scale-down studies for assessing the impact of different stress parameters on growth and product quality during mammalian cell culture. In: Proceedings of the 14th European Conference On Mixing. pp. 10–13.

[bib48] Sengupta N., Rose S.T., Morgan J.A. (2011). Metabolic flux analysis of CHO cell metabolism in the late non-growth phase. Biotechnol. Bioeng..

[bib49] Sharp K.V., Adrian R.J. (2001). PIV study of small-scale flow structure around a Rushton turbine. AIChE J..

[bib50] Smales C.M., James D.C. (2005). Therapeutic Proteins: Methods and Protocols.

[bib51] Sorg R., Tanzeglock T., Soos M., Morbidelli M., Périlleux A., Solacroup T., Broly H. (2011). Minimizing hydrodynamic stress in mammalian cell culture through the lobed Taylor-Couette bioreactor. Biotechnol. J..

[bib52] Terrier B., Courtois D., Henault N., Cuvier A., Bastin M., Aknin A., Dubreuil J., Petiard V. (2006). Two new disposable bioreactors for plant cell culture: the wave and undertow bioreactor and the slug bubble bioreactor. Biotechnol. Bioeng..

[bib54] Zhang X., Stettler M., De Sanctis D., Perrone M., Parolini N., Discacciati M., De Jesus M., Hacker D., Quarteroni A., Wurm F. (2010). Use of orbital shaken disposable bioreactors for Mammalian cell cultures from the milliliter-scale to the 1000-liter scale. Adv. Biochem. Eng. Biotechnol..

[bib55] Zhou G., Kresta S.M. (1996). Impact of tank geometry on the maximum turbulence energy dissipation rate for impellers. AIChE J..

[bib56] Zhu H., Nienow A.W., Bujalski W., Simmons M.J.H. (2009). Mixing studies in a model aerated bioreactor equipped with an up- or a down-pumping “Elephant Ear” agitator: power, hold-up and aerated flow field measurements. Chem. Eng. Res. Des..

